# Unveiling the impact of date-specific analytics on vehicle fuel consumption and emissions: A case study of Shiraz city

**DOI:** 10.1016/j.heliyon.2024.e36713

**Published:** 2024-08-22

**Authors:** Elmira Bagheri, Masoud Masih-Tehrani, Mohammad Azadi, Ashkan Moosavian, Sarah Sayegh, Mansour Hakimollahi

**Affiliations:** a School of Automotive Engineering, Iran University of Science and Technology, 16846-13114, Iran; b Faculty of Mechanical Engineering, Semnan University, 35131-19111, Iran; c Department of Agricultural Engineering, Technical and Vocational University (TVU), 1435661137, Iran; d Faculty of Mechatronic Engineering, Ahlul-Bayt International University, 1955883133, Iran

**Keywords:** Date-specific, Emissions, Fuel consumption, Real driving cycle, Vehicle simulation

## Abstract

Understanding the factors affecting internal combustion engine performance is crucial for improving emissions and fuel efficiency in real traffic. This paper investigates the influence of date-specific factors, such as working days and holidays on fuel consumption and emissions of a representative internal combustion engine in the context of Shiraz city. The data was collected by measuring the speed of vehicle on a specific route during peak traffic times on both working days and holidays. The determined route was calculated by a qualitative and quantitative method. K-means clustering and principal component analysis are employed to design representative driving cycles, then combining micro-trips and smoothing them to develop driving cycles. Characteristics of both driving cycles, emissions such as HC, NOx, CO, and fuel consumption were specified under a simulated vehicle model of Peugeot 206 and placed through both real driving cycles by using advanced vehicle simulation software. It has been observed that variations in fuel consumption and emissions between holidays and working days can be attributed to distinct driving patterns and characteristic parameters, such as acceleration, speed, and driving time. Contrary to common assumptions, the study found that fuel consumption was approximately 8 % higher on holidays compared to working days, primarily due to increased driving time and higher average speeds. Moreover, higher acceleration and speed on holidays led to a significant increase in CO emissions (about 36 %) and NOx emissions (about 4 %) compared to working days. However, HC emissions was found to be 27.03 % higher on working days according to lower speed. This study helps in reducing fuel consumption and emissions by uncovering internal combustion engine factors, optimizing driving patterns, and promoting sustainable transport on working days and holidays.

## Highlights

• Collecting data from routes in both working days and holidays during peak hours in Shiraz city.
• Utilization of the Analytic Hierarchy Process (AHP) for selecting test route.
• Using K-means clustering and PCA algorithms to design real driving cycles.
• Considering 12 characteristic parameters in generating each drive cycle.
• Deriving HC, CO, NOx emissions and fuel consumption via ADVISOR simulation software.

## 1 Introduction

The driving cycle is a sequence of speed and time measurements tailored for specific vehicles in a particular region. There is an experiment to investigate the impact of emissions and fuel consumption. The drive cycle is frequently utilized in vehicle simulation to predict the behavior of different powertrain configurations [1]. Any type of vehicle with Internal Combustion Engines (ICEs) has been a significant contributor to the release of greenhouse gas emissions since they dominate the use of the transportation sector and have a large market share [2]. Nowadays, government agencies have implemented stricter emission regulations with national or international standards that introduce emission threshold levels and methods of measuring them. These legal requirements have forced vehicle manufacturers to look for new technologies to meet the standards [3]. In this regard, emission assessment is important for conventional vehicle operations. The measurement of vehicle exhaust emissions is mainly divided into two methods: real-world measurement and laboratory measurement [4].

Real-world emissions measurements are performed by collecting real-time emissions data under road traffic conditions at specific locations. Although the measurement method reflects the actual amounts of emissions, it is an expensive method due to the various types of vehicles and the limitations of operating conditions. However, measurements in on-road traffic conditions can also be essential for use in emission factor data for laboratory measurements and the development of test methods [5]. Laboratory tests do not adequately reflect real driving conditions on the road. Therefore, the approach for evaluating greenhouse gas emissions from vehicles is to collect data under real conditions in driving environments. With the data, emissions are more accurately assessed under a wide range of environmental conditions during on-road operations. In this regard, several emission estimation tools have been developed considering several factors affecting vehicle emissions, such as COPERT, ARTEMIS, and MOVES [6].

The emissions and fuel consumption of vehicles are commonly impacted by factors such as road geometry, ambient temperature, route selection, fuel type, traffic management techniques [7], and vehicle attributes, including weight [8]. Triantafyllopoulos et al. [9], discovered that engaging in dynamic driving on an ascending roadway led to eightfold and threefold increase in CO_2_ and NOx emissions, respectively. According to the findings of Pavlovic et al. [10], the influence of road incline on fuel consumption difference is commonly more than 50 % when comparing segments featuring varying road gradients below −1 % to segments with grades above +1 %. On the other hand, in urban areas, the act of starting a vehicle from a cold state can make a meaningful contribution to both overall emissions and fuel consumption [11]. This is primarily attributed to the prevalence of short journeys and frequent engine initiations [12]. Dropping the ambient temperature ranging from 77 °F to 46.4 °F within the initial 300-s period led to an increase in CO_2_, a surge in CO, and a decline in NOx [13]. When conducting a Real Driving Emission (RDE) test on diesel vehicles, journeys in the temperature range of 41 °F to 50 °F exhibit variances of up to 30 % in NOx emissions, whereas the difference is not as significant for petrol/gasoline vehicles [14].

Regarding the RDE regulations imposed by the European Union, the distribution of rural roads, urban roads, and highways is roughly equal, yet they have varying impacts on emission levels and fuel consumption [15]. Generally, earlier studies relied on subjective judgments for route selection, considering factors like home-to-work trips, population densities, and road classifications. These evaluations were qualitative, necessitating a more systematic approach. For instance, Liu et al. [16] collected data from a taxi area in Tianjin, China, covering various road types and areas. Study [17] focused on Hefei city, selecting typical roads to obtain driving speed and range data for electric vehicles. However, Zhao et al. [18] investigated the traffic situation in Xi'an city by calculating urban road network metrics and traffic flow, ultimately designing a 38.4 km circular route for the electric vehicle driving cycle test and achieving comprehensive results.

In a study, Williams et al. [19], carried out RDE performance assessments on three different examination routes. The first route primarily consisted of urban driving sections, with no inclusion of an expressway section. The second route predominantly represented driving on rural roads, while the last route adhered to the regulations outlined in the European RDE legislation. Their findings revealed a direct correlation between the duration of the test and the increase in CO emissions, irrespective of the particular category of examination route implemented. In those tests, they acquired elevated levels of CO and HC compared to the levels observed in the European RDE examination [20].

The behavior of drivers is another important factor that indirectly has a significant effect on fuel consumption and greenhouse gas emissions of vehicles [21]. The use of eco-driving techniques in smart transportation has recently received much attention. Eco-driving offers suitable solutions to reduce traffic, control fuel consumption, and reduce emissions from vehicle exhaust. To reduce fuel consumption in Ref. [22], a new design for determining the appropriate speed at signalized intersections has been presented. In that study, the approximate time of the car crossing the intersection was estimated with the help of the Gaussian process regression model. Bakibillah et al. [23] presented a dynamic eco-driving system consisting of predictive control and fuzzy logic, which can reduce CO emissions and fuel consumption on steep roads. Other examples of existing methods for controlling vehicle emissions and fuel consumption on hilly roads and under a mixed traffic environment can be seen in Ref. [24]. Recently, Shahariar et al. [25] studied the effect of driving style on fuel consumption and pollutant gases produced from diesel and diesel-biodiesel blend. Including peak and off-peak traffic in the scenarios defined on a turbocharged diesel engine is one of the prominent features of this study. The results showed that the growth in load influenced by the sudden change in acceleration caused the off-peak traffic to show more pollutants than the peak traffic.

In addition to the previously mentioned factors, several studies have investigated the impact of specific dates on fuel consumption and emissions in various regions. By taking these factors into account, it may be possible to improve fuel efficiency and reduce vehicle emissions to promote a more sustainable environment. For instance, Du et al. [26] developed a model based on Back Propagation Neural Network (BPNN) to forecast the fuel consumption patterns on working days, public holidays, and weekends. This study involved over 13k cars in Beijing and considered parameters such as speed, fuel consumption, driver information, and road conditions. The results revealed that the highest fuel consumption on working days, averaging 5.8 l/100 km, occurred on Fridays, while the average fuel consumption on weekends was lower than on working days. Furthermore, Wang et al. [27] conducted a case study in China to investigate holiday travel and the associated challenges for transportation infrastructure. They explored the relationship between energy consumption, travel activities, and greenhouse gas emissions during holidays, and proposed potential solutions. Various modes of transportation, including buses, subways, and cars, were examined in this study. In another research study [28], conducted in Beijing, the relationship between energy consumption, traffic patterns, and pollutant emissions on weekdays, weekends, and holidays was analyzed. The authors demonstrated that energy consumption is higher on holidays, while gas emissions are lower compared to weekdays and weekends. For instance, it was found that energy consumption significantly increases before 11 a.m. on weekends and holidays due to the influx of people heading to parks for recreational activities.

While previous research has explored the impact of specific dates on fuel consumption and emissions, few studies have specifically examined how these days affect fuel consumption and emissions within the context of driving cycles and their characteristics. This paper focuses on creating a domestic driving cycle consisting of 12 parameters that encompass private vehicles. It also investigates the impact of date-specific factors, such as working days and holidays, on characteristic parameters, fuel consumption, and emissions. The outcomes of the research are expected to reveal disparities in performance across specific dates, providing valuable insights for the development of efficient eco-driving initiatives and devices. Given the substantial variations in driving patterns across different areas and the uniqueness of each region's driving cycle [29], this study focuses on Shiraz city.

The innovations of the present work can be summarized as.

• Providing a 25.9 km test route consisting of different types of highways, main roads, and secondary road under different traffic conditions by using the Analytic Hierarchy Process (AHP).
• Calculation real driving cycles by considering 12 characteristic parameters with the help of K-means clustering algorithms and Principal Component Analysis (PCA).
• Determining the amount of fuel consumption and emissions according to the characteristic parameters of drive cycles while providing a comprehensive comparison of them on holidays and working days.

The rest of the paper is modified as follows. In section 2, the proposed methodology is demonstrated in four stages. The first stage outlines details regarding route selection. Data collection and vehicle testing are described in the subsequent stage. The third and fourth stages showcase how to calculate characteristic parameters and analyze data using the clustering method, respectively. Section 3 focuses on the discussion and results of driving cycles, characteristic parameters, validation, fuel consumption, and emissions. Finally, section 4 includes the conclusion.

## 2 Materials and methods

**Fig. 1:**
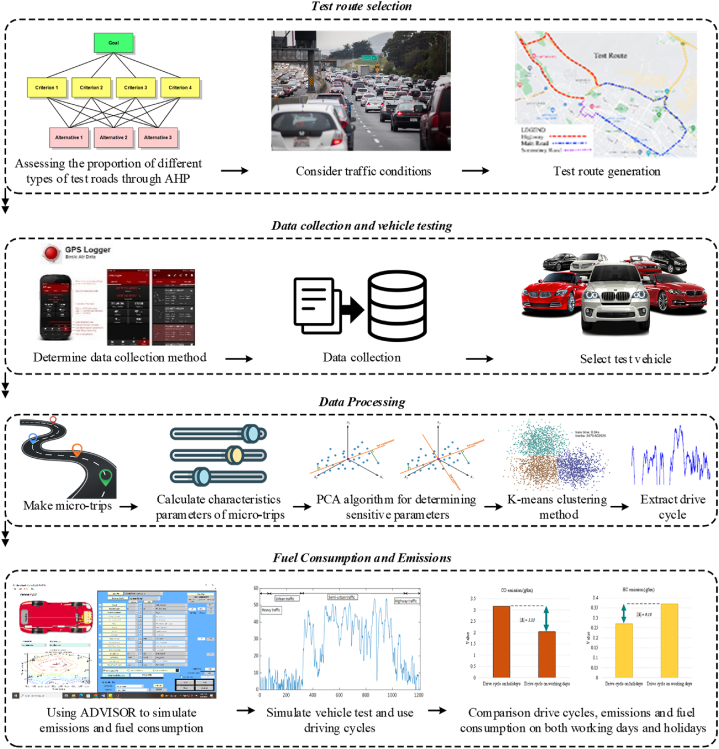
The flowchart of the research method.

The process is visualized through a diagram in Fig. 1. The subsequent sections offer comprehensive step-by-step instructions.

### 2.1 Test route selection

Traffic congestion within urban areas is known to fluctuate based on time and route, with more pronounced congestion expected on typical working days and peak hours, in contrast to public holidays, weekends, and off-peak hours [30]. In the designated study area, it is crucial to select representative routes that take into account factors such as road types, topology, crossroads, population density, slope, and weather circumstances. All of these factors can impact traffic movement and are considered during the test route selection process.

In this research, factors such as the proportion of test route types, traffic conditions, and peak traffic hours were carefully considered in the selection of the test route. The allocation of various test route types was determined using AHP. By utilizing AHP, this study seeks to improve the objectivity and reliability of the route selection process, leading to a more thorough and informed evaluation of the impacts of different route types.

This study focuses on Shiraz city. Shiraz is the fifth-most populous city in Iran, is the capital of Fars Province, situated in the southwest amidst the Zagros Mountains. Covering 177 sq. km, it experiences hot summers and mild winters. Surrounded by mountains and plains, Shiraz has a mix of road networks, public transit, and private vehicles. Its transportation infrastructure includes highways, urban roads, and a Bus Rapid Transit (BRT) system. The growing population and vehicle numbers contribute to traffic congestion and environmental concerns, necessitating study and solutions amid global warming and greenhouse gas emissions.

The initial step is to create a hierarchical framework. In the modeling phase, the decision-making objective is defined by organizing decision elements into an interrelated hierarchy. The problem of determining the distribution of various types of test route is divided into three hierarchies: goal, criteria, and scheme. Determining the proportion of test route is considered a decision-making goal, as illustrated in Table 1. The criteria examined to evaluate the types of test routes include familiarity, economy, convenience, driving time, traffic conditions, road conditions, driving distance, and road slope. These factors determine the type of test route that is important. The paper considers highways, main roads, and secondary roads as types of decision schemes.

**Table 1:** The hierarchical structure model.

Goal	The distribution of various test route types							
Criteria	Familiarity	Economy	Convenience	Driving time	Traffic conditions	Road conditions	Driving distance	Road slope
Schemes	Highway		Main Road			Secondary Road		

The following step involves the creation of a judgment matrix. This matrix is developed by evaluating the significance of each element in the hierarchy relative to the upper hierarchy. To generate the judgment matrix, questionnaires were designed to convert the personal opinions and practical knowledge of decision-makers into numerical matrices. In this study, decision-makers were randomly selected from the population of Shiraz city residents using the simple random sampling method. The sample set comprised 45 participants, selected from the population, considering the study's time and cost limitations. To prevent potential bias, the Statistical Process Control (SPC) concept [31] monitored the sample selection process. This allowed for the removal of samples with extreme variations, such as decision-makers with unusually young or old age values. As a result, adequacy of the sample size for generating the judgment matrix was deemed statistically acceptable, and the findings could be generalized to the entire region under study.

**Fig. 2:**
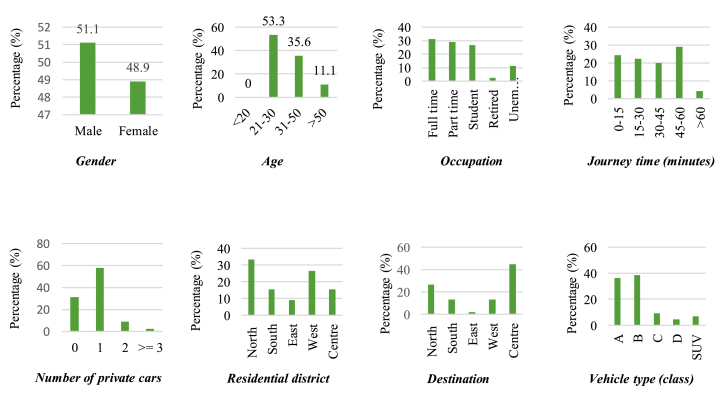
The characteristics of the decision-makers.

Fig. 2 illustrates the characteristics of the decision-makers, including their gender, age, employment status, vehicle ownership, vehicle type (by considering the volume and size of the vehicles), area of residence, the destination of most trips, and the duration of their trips per day.

For this hierarchical structure model, the questionnaire consisted of a total of 52 questions. The questionnaire relies on evaluating every pair of factors within the criterion hierarchy [18]. The judgment matrix A (i.e., the criteria matrix in relation to the goal matrix) is determined in Equation (1) and demonstrated in Equation (2).

In Equation (1), $a_{ij}$ represents the ratio of the importance of criterion i to the importance of criterion j. For creating the judgment matrix A, decision-makers responded to 28 questions concerning the hierarchy of criteria in relation to the goal. The average of the responses from these decision-makers was then used to construct the judgment matrix A. The relative importance of these two factors is also assessed across nine levels $\left(d_{ij}=5,4,...,1,...,1/4,1/5\right)$.

The values in the judgment matrix represent the relative importance of different criteria in relation to each other. For example, the number specified in Equation (2) demonstrates the importance of familiarity compared to economy conditions when deciding the type of test route. Similarly, the numbers located on the main diagonal of the matrix represent relationships between each criterion and itself. Calculations for other criteria in the hierarchy were also conducted in the same way.

The judgment matrices i.e., $D_{1},D_{2},...,D_{8}$ which compare the schemes hierarchy to the criterion hierarchy, were derived using the same methodology as judgment matrix A.

(1) $$A={\left(a_{ij}\right)}_{n\times n,}a_{ij}>0,a_{ij}=\frac{1}{a_{ji}}\left(i,j=1,2,...,n\right)$$

(2) $$\begin{array}{l} \begin{array}{cccc} \;\text{Familiarity} & \text{Economy} & \;...\; & \;\text{Road}\;\text{slope} \end{array}\\ A=\begin{array}{c} \;\text{Familiarity}\\ \;\text{Economy}\\ \;...\\ \;\text{Road}\;\text{slope} \end{array}\;\left[\begin{array}{cccccccc} 1 & \;2\; & \;2\; & \;2\; & \;1\; & \;1\; & \;2\; & \;1\;\\ 1/2 & 1 & 1 & 1 & 1/2 & 1 & 1 & 1\\ 1/2 & 1 & 1 & 1 & 1/2 & 1 & 2 & 1\\ 1/2 & 1 & 1 & \;1\; & 1 & 1 & 1 & 1\\ 1 & 2 & 2 & 1 & 1 & 3 & 3 & 3\\ 1 & 1 & 1 & 1 & 1/3 & 1 & 2 & 1\\ 1/2 & 1 & 1/2 & 1 & 1/3 & 1/2 & 1 & 1\\ 1 & 1 & 1 & 1 & 1/3 & 1 & 1 & 1 \end{array}\right] \end{array}$$

The judgment matrices $D_{1},D_{2},...,D_{8}$ are shown as Equation (3).

(3) $$\begin{array}{l} D_{1}=\left[\begin{array}{ccc} 1 & 1 & 2\\ 1 & 1 & 1\\ 1/2 & 1/2 & 1 \end{array}\right],D_{2}=\left[\begin{array}{ccc} 1 & 2 & 3\\ 1/2 & 1 & 2\\ 1/3 & 1/2 & 1 \end{array}\right],D_{3}=\left[\begin{array}{ccc} 1 & 2 & 2\\ 1/2 & 1 & 2\\ 1/2 & 1/2 & 1 \end{array}\right],D_{4}=\left[\begin{array}{ccc} 1 & 1 & 2\\ 1 & 1 & 1\\ 1/2 & 1 & 1 \end{array}\right]\\ D_{5}=\left[\begin{array}{ccc} 1 & 2 & 2\\ 1/2 & 1 & 2\\ 1/2 & 1/2 & 1 \end{array}\right],D_{6}=\left[\begin{array}{ccc} 1 & 2 & 2\\ 1/2 & 1 & 2\\ 1/2 & 1/2 & 1 \end{array}\right],D_{7}=\left[\begin{array}{ccc} 1 & 1 & 2\\ 1 & 1 & 1\\ 1/2 & 1 & 1 \end{array}\right],D_{8}=\left[\begin{array}{ccc} 1 & 2 & 3\\ 1/2 & 1 & 2\\ 1/3 & 1/2 & 1 \end{array}\right] \end{array}$$

The next step involves evaluating the consistency of judgment matrices. A consistency ratio is a tool that determines the consistency of judgments [18]. If the consistency ratio for the matrix of pairwise comparisons is greater than 0.1, the preference judgments made have inconsistency, and this inconsistency should be resolved [32].

The fourth step is to compute the significance of each scheme in relation to the overarching objective, as demonstrated in Equation (4). In summary, the operation consists of the following steps.

1. Summing up the values in each column of the judgment matrix, dividing each value by the sum of its respective column, and generating a new matrix referred to as the normalized comparison matrix.
2. Computing the mean of each row in the normalized comparison matrix. The average calculated for each row in the matrix indicates the importance of the decision elements that correspond to those rows.
3. Finally, to determine the values or rankings of the schemes, weight matrices of $D_{1},D_{2},...,D_{8}$ is multiplied by a weight vector A [33]. (In Equation (4), $W_{0},W_{1},...,W_{7}$, and $W_{8}$ are weight matrices of $A,D_{1},D_{2},...,D_{8}$, respectively.)

(4) $$W=W_{0}{\left[\begin{array}{c} W_{1}^{T}\\ W_{2}^{T}\\ W_{3}^{T}\\ W_{4}^{T}\\ W_{5}^{T}\\ W_{6}^{T}\\ W_{7}^{T}\\ W_{8}^{T} \end{array}\right]}^{T}={\left[\begin{array}{ccc} 0.411 & 0.327 & 0.261\\ 0.538 & 0.296 & 0.163\\ 0.49 & 0.311 & 0.197\\ 0.411 & 0.327 & 0.261\\ 0.49 & 0.311 & 0.196\\ 0.49 & 0.311 & 0.196\\ 0.411 & 0.327 & 0.261\\ 0.538 & 0.296 & 0.163 \end{array}\right]}^{T}\times \left[\begin{array}{c} 0.169\\ 0.096\\ 0.106\\ 0.109\\ 0.216\\ 0.112\\ 0.08\\ 0.103 \end{array}\right]=\left[\begin{array}{c} 0.47\\ 0.32\\ 0.21 \end{array}\right]$$

**Fig. 3:**
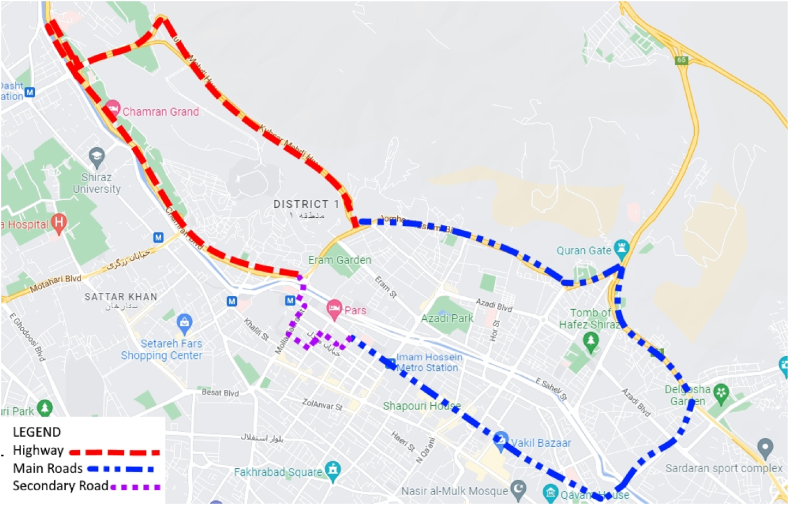
The representative of the test route.

Traffic conditions are often selected as one of the criteria for choosing driving routes during data collection. In this case, the online traffic site of Shiraz city was monitored for a week, including working days and holidays, at various times. During this survey, areas with higher traffic flow and peak traffic hours were identified. Moreover, according to research [34], the highest concentration of pollutants occurs during the hours of 9–11 in the morning and 6–8 in the evening. Online traffic sites also confirm the highest congestion during these hours, validating the results. Based on the obtained information, routes are suggested to comprise 47 % highway, 32 % main road, and 21 % secondary road. A test route is suggested based on the obtained results. The length of the proposed route is 25.9 km. The distribution and length of each type of test route are presented in Table 2. The proposed test route is displayed in Fig. 3.

**Table 2:** The proportion and distances of the test route.

*Type of Roads*	Contribution to the AHP (%)	Contribution to the test route (%)	Length of test route (km)
Highway	47	47	12.2
Main road	32	33.2	8.6
Secondary road	21	19.6	5.1

### 2.2 Data collection

Based on the available vehicles in the specified study area, it is essential to select representative vehicles and equip them with instruments capable of collecting and storing data on driving activities along the chosen routes.

Real-world driving cycle data of Shiraz were collected on working days and holidays, considering peak traffic hours, along a selected route that includes the town centre, residential area, and highway, representing mostly city activities (as shown in Fig. 3). The slope of the route is negligible [35].

In this research, the data on the vehicle's speed is continuously recorded at any moment using a GPS logger program installed on a mobile phone. The data collection frequency in this method is 1 Hz. GPS logger is a straightforward program that records the position, speed, altitude, direction, and numerous trip statistics. Additionally, it allows access to the list of recorded trips at any time. The program generates output in GPX, KML, and TXT formats.

Data collection was done by a driver who is familiar with the roads of Shiraz, 27 years old, and has 4 years of driving experience. Utilizing the same driver with a familiarity of the local roads ensures a comprehensive comprehension of driving conditions, thereby enhancing the accuracy and consistency. The GPS data collection for the study lasted for a total of 4 h and 30 min. The basis for choosing this time period was to cover a sequence of various events, regardless of the distance traveled. Among these events, we can mention low speed trips, cold starts, and driving in different traffic congestions. Taking these into account makes the collected data a valid representation of different driving conditions. In terms of time distribution, approximately 2 h and 45 min were dedicated to weekdays, while the remaining duration was allocated to normal Fridays, which are public holidays in Iran.

This data was gathered during clear weather conditions to mitigate the impact of adverse weather. Furthermore, data was not collected under unusual route conditions or traffic situations. The information utilized in this research has been obtained from a Peugeot 206 vehicle, which is among the most prevalent vehicles in Iran and has structural characteristics that are comparable to the present top-selling automobile in the region [36,37]. A survey involving 712 volunteer drivers [38] found that this vehicle was the second most popular vehicle, while another study [39] reported a market share of 10.5 % among domestic vehicles, ranking second by a small margin behind the top-selling vehicle. The technical specifications of this vehicle are given in Table 3.

**Table 3:** The technical specifications of the vehicle [40].

Parameters	Values
Overall height (m)	1.453
Overall length (m)	4.292
Overall width (m)	1.684
Wheelbase (m)	2.445
Front-track (m)	1.457
Rear-track (m)	1.448
Weight (kg)	1100
Engine power (kw)	78
Torque (nm)	142
Max speed (km/h)	193
Fuel consumption in mixed driving (1/l00 km)	6.6
Acceleration 0–100 (s)	11.4

### 2.3 Characteristics of acquired data and principal component analysis

After collecting data on the test route under real-world conditions, the following stage is to calculate the characteristic parameters from the obtained data. The purpose of this work is to determine the sensitivity parameters. Additionally, these parameters are employed to compare the characteristic parameters of real driving cycles on both working days and holidays along a particular route.

In the evaluation stage, twelve characteristic parameters, such as driving time percentage, standing time percentage, cruise time percentage, accelerating time percentage, decelerating time percentage, average driving speed, average trip speed, maximum speed, maximum positive/negative acceleration, and the standard deviation of speed/acceleration, were selected [41]. Due to excessive number of characteristic parameters, the analysis becomes more complicated [42]. To simplify the analysis of the characteristic parameters, PCA was employed for dimension reduction.

Before applying the PCA method, it is crucial to ensure that the collected data volume is adequate for this technique. Two common methods to assess this are the Kaiser-Meyer-Olkin (KMO) test and Bartlett's test. The KMO index is a positive real value ranging from 0 to 1, with larger values indicating a stronger predictive relationship between variables and a smaller total error. A KMO value close to 0 suggests that the sample is unsuitable for PCA, while values between 0.5 and 0.7 are acceptable with caution [43].

Bartlett's test is another method to check the adequacy of data by examining the existence of a significant correlation between variables and determining if the correlation matrix is a unit matrix. Using IBM Statistics SPSS 27, the results for these tests on the data in question are as follows: The final KMO index value is 0.682, which guarantees the suitability of factor analysis on the data. Additionally, the Bartlett's test of sphericity is significant (p < 0.001) with degrees of freedom equal to 66, allowing us to reject the null hypothesis [43].

PCA is a statistical technique that identifies a reduced set of principal components from the original set of factors, eliminating unnecessary information [44]. The previously mentioned twelve characteristic parameters are employed to establish a new comprehensive evaluation, as demonstrated in Equation (5). The equation employs normalized kinematic characteristic parameters denoted as $x_{1},x_{2},...,x_{12}$ along with twelve comprehensive indexes represented by $y_{1},y_{2},...,y_{12}$ and coefficients of kinematic characteristic parameters $a_{1},a_{2},...,a_{12}$ to establish a mathematical relationship.

(5) $$\left\{ \begin{array}{c} y_{1}=a_{11}x_{1}+a_{12}x_{2}+...+a_{112}x_{12}\\ y_{2}=a_{21}x_{1}+a_{22}x_{2}+...+a_{212}x_{12}\\ \;...\\ y_{12}=a_{121}x_{12}+a_{122}x_{12}+...+a_{1212}x_{12} \end{array}\right.$$

After performing an orthogonal transformation from the PCA algorithm, the data was analyzed as Equation (6) [18].

(6) $$\left\{ \begin{array}{c} y_{1}^{\prime }=L_{1}^{T}X=l_{11}x_{1}+l_{12}x_{2}+...+l_{112}x_{12}\\ y_{2}^{\prime }=L_{2}^{T}X=l_{21}x_{1}+l_{22}x_{2}+...+l_{212}x_{12}\\ \;...\\ y_{12}^{\prime }=L_{12}^{T}X=l_{121}x_{12}+l_{122}x_{12}+...+l_{1212}x_{12} \end{array}\right.$$

The twelve comprehensive indexes, denoted as $y_{1}^{\prime },y_{2}^{\prime },...,y_{12}^{\prime }$, are mutually independent and designated as the first, second, …, and twelfth principal components. The eigenvectors that match the decreasing order of eigenvalues $\lambda_{1},\lambda_{2},...,\lambda_{12}$ (where $\lambda_{1}\geq \lambda_{2}\geq ...\geq \lambda_{12}$) are $l_{1},l_{2},...,l_{12}$, and they are unit vectors [18]. Equations (7), (8) calculate the variance contribution ratio of individual principal components and the cumulative variance contribution ratio, respectively. In Equations (7), (8), the variable ' i' ranges from 1 to p, with p being equal to 12.

(7) $$P=\frac{l_{i}}{\sum_{k=1}^{p}l_{k}}\times 100\%,i=\left(1,2,...,p\right)$$

(8) $$CP=\frac{\sum_{k=1}^{i}\lambda_{i}}{\sum_{k=1}^{p}\lambda_{k}}\times 100\%,i=\left(1,2,...,p\right)$$

**Fig. 4:**
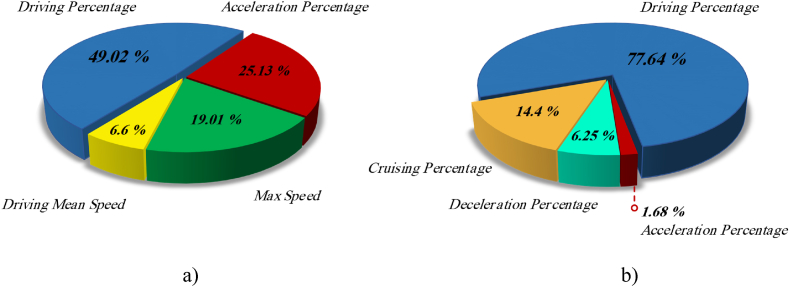
Variance percentage of characteristics parameters on (a) holidays and (b) working days.

Following the application of PCA, the initial pair of principal components with the highest variance percentage were chosen as the characteristic parameters for performing clustering analysis [45], similar to reference [46]. These characteristic parameters are called sensitive parameters. Fig. 4a and Fig. 4b represent the variance percentage of characteristic parameters on holidays and working days, respectively.

As shown in Fig. 4a, the driving percentage with 49.02 % and the accelerating percentage with 25.13 % were selected as sensitive parameters on holidays. In addition, the driving percentage with 77.64 % and the cruising percentage with 14.4 % were chosen as sensitive parameters on working days, as shown in Fig. 4b.

### 2.4 Data analysis using K-means clustering method

This study utilizes the K-means clustering method for micro-trip segmentation due to its simplicity and effectiveness in managing large datasets [47].

Cluster analysis is employed to categorize structures exhibiting common attributes into one class and structures with different characteristics into another class. This study shares similarities with the classifications discussed in Refs. [44,48], as it categorizes driving patterns into four distinct groups: heavy, urban, semi-urban, and highway traffic conditions as interpreted cluster 1, cluster 2, cluster 3, and cluster 4, respectively.

**Fig. 5:**
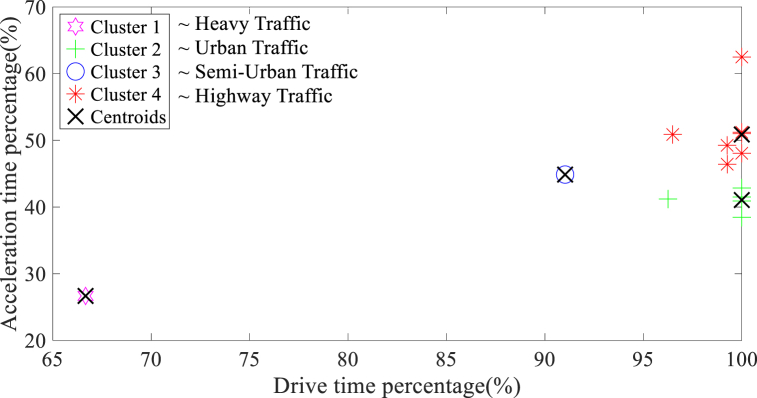
The K-means clustering findings derived from sensitive parameters on holidays.

**Fig. 6:**
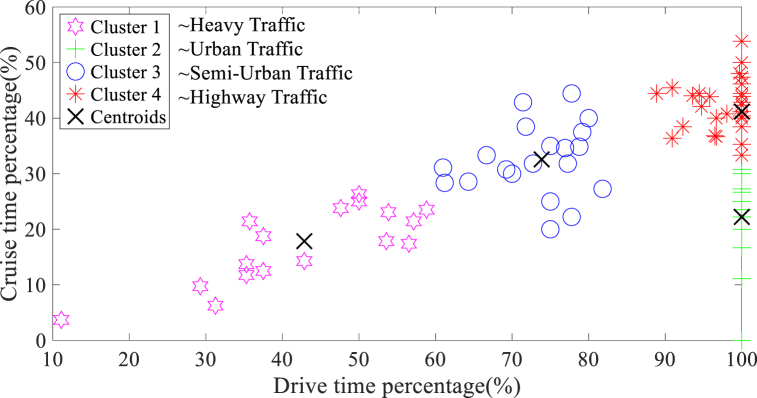
The K-means clustering findings derived from sensitive parameters on working days.

The results of the K-means clustering analysis, which considered sensitive parameters on holidays and working days are indicated in Fig. 5, Fig. 6, respectively. The increased sensitive parameters on working days, as evident in the clustering analysis, is likely attributed to the higher quantity of micro-trips due to frequent braking and starting in traffic conditions.

For K-means clustering, the squared Euclidean distance shown in Equation (9) is the most commonly used method. Generally, the data was analyzed using squared Euclidean distance [49]:

(9) $$d_{ij}=\sum_{k=1}^{p}{\left(x_{ik}-x_{jk}\right)}^{2}$$

Each micro-trip has p parameters, including $d_{ij}$, the distance of each micro-trip, and $x_{ik}$, the micro trip of number i in parameter k.

The methodology for developing a driving cycle is a multi-step process, commencing with the segmentation of original data into micro-trips, followed by the calculation of their characteristics, identification of sensitive parameters using PCA, and culminating in the selection of the optimal number of micro-trips to form the driving cycle. Most steps are accomplished by using a specially coded MATLAB program.

## 3 Results and discussions

### 3.1 Driving cycles and characteristic parameters

To derive driving cycles, it is essential to identify micro-trips that characterize each cluster. A driving cycle that accurately represents the driving patterns is constructed by selecting micro-trips that are nearest to the cluster centers. This process continues until the time-sharing criterion is met.

**Fig. 7:**
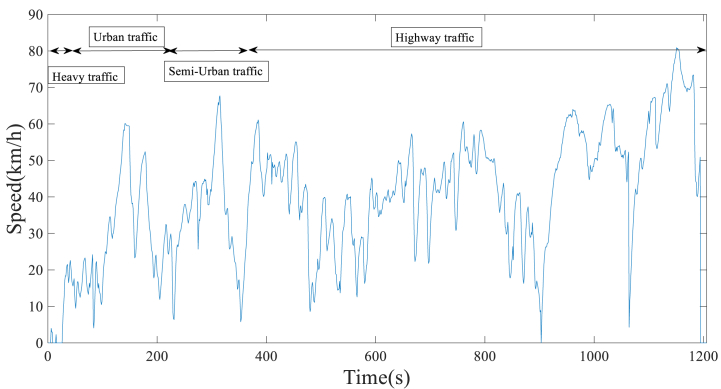
Developed Shiraz driving cycle on holidays.

**Fig. 8:**
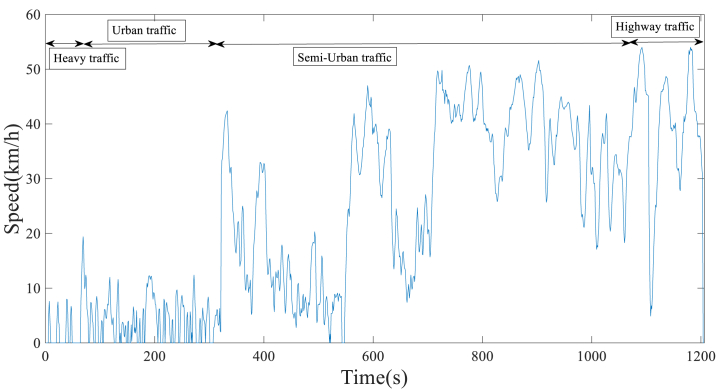
Developed Shiraz driving cycle on working days.

The length of the driving cycle plays a crucial role in ensuring accurate representation and improved measurement of emission levels and fuel consumption during the testing process. The testing process should ideally be efficient and straightforward, without excessive duration or complexity [50]. In general, the duration of standard driving cycles and representative real-world driving cycles is between 600 and 1800 s [45]. This paper considered 1200 s for time sharing. The driving cycles of Shiraz city on both holidays and working days are obtained in Fig. 7, Fig. 8, respectively. The lower speed of working days driving cycles, compared to holiday driving cycles, indicates that traffic congestion is more prevalent during weekdays.

In cycle evaluation, characteristic parameters are used to verify that the developed cycle accurately represents real-world driving patterns [51]. The characteristics of the real driving cycles on both working days and holidays were calculated, as presented in Table 4. As demonstrated in Table 4, the driving cycle on working days has a higher standing percentage of 8.32 % compared to the holiday driving cycle, suggesting heavier traffic conditions. Furthermore, comparing the deceleration time percentage of approximately 35.52 % during working days to 31.56 % during holidays indicates that the driver applies more braking on working days. Moreover, during holidays, with an acceleration time of 49.50 %, a standard deviation of acceleration of around 0.86, and a standard deviation of speed of approximately 43.16 km/h, it is observed that driving is more aggressive on holidays [51].

**Table 4:** Characteristics of driving cycles on holidays and working days.

Parameters	Holidays	Working days
Duration (s)	1204	1202
Distance (km)	21.1	12.5
Driving time (s)	1172	1102
Cruise Time (s)	196	207
Standing time (s)	32	100
Drive time spent decelerating (s)	380	427
Drive time spent accelerating (s)	596	468
Percent of time driving (%)	97.34	91.68
Percent of time accelerating (%)	49.50	38.93
Percent of time cruising (%)	16.27	17.22
Percent of time decelerating (%)	31.56	35.52
Percent of time standing (%)	2.65	8.32
Average driving speed (km/h)	40.46	25.42
Average trip speed (km/h)	39.64	23.29
The standard deviation of speed (km/h)	43.16	28.30
Maximum speed (km/h)	80.89	54.00
Average negative acceleration (m/s2)	−0.62	−0.46
Average positive acceleration (m/s2)	0.42	0.43
The standard deviation of acceleration (m/s2)	0.86	0.59

### 3.2 Validation of the construction method

The developed driving cycle must undergo a thorough evaluation to ensure its alignment with the collected on-road data, enabling a meaningful comparison against the standard driving cycle and identifying any deviations.

To validate the advanced and scientific attributes of the proposed method for constructing driving cycles, a comparison was made between the created driving cycles and two standard driving cycles. Furthermore, the validation of the characteristic parameters was conducted using the Root Mean Square Error (RMSE) and standard deviation metrics [52], ensuring a comprehensive and accurate evaluation of the proposed method. By comparing the FTP-72 [53] and Mashhad [45] standard driving cycles with the developed driving cycles shown in Table 5, several noteworthy differences can be observed. The primary reasons for using these urban-based standard cycles and the Mashhad driving cycle, which is a prevalent driving cycle in Iran, are to provide a basis for comparison.

**Table 5:** Comparisons of developed driving cycles and standard driving cycles.

Parameters	Holidays	Working days	FTP-72	Mashhad
Percent of time driving (%)	97.34	91.68	86.19	89.70
Percent of time accelerating (%)	49.50	38.93	36.96	40.23
Percent of time cruising (%)	16.27	17.22	18.04	4.01
Percent of time decelerating (%)	31.56	35.52	31.19	55.76
Percent of time standing (%)	2.65	8.32	13.81	10.30
Average trip speed (km/h)	39.64	23.29	31.60	28.36
Average driving speed (km/h)	40.46	25.42	36.60	29.84
The standard deviation of speed (km/h)	43.16	28.30	21.46	3.14
Maximum speed (km/h)	80.89	54.00	91.15	78.01
Average negative acceleration (m/s2)	−0.62	−0.46	−0.46	−0.70
Average positive acceleration (m/s2)	0.42	0.43	0.43	0.66
The standard deviation of acceleration (m/s2)	0.86	0.59	0.64	0.53

Upon examining the driving time percentage, the developed driving cycles reveal longer driving times compared to the FTP-72, although the working days' driving time slightly deviates from the Mashhad driving cycle. The developed cycles exhibit similar cruising durations as the FTP-72 cycle, while this parameter is notably lower in Mashhad.

The acceleration time percentage is higher in the Shiraz driving cycle during holidays compared to the other cycles. This observation can be explained by the reduced traffic, which allows for more acceleration opportunities. In contrast, the high percentage of decelerating time on working days compared to other cycles signifies the influence of heavy traffic, leading to more braking incidents.

When comparing the speeds of the four driving cycles, it is noticeable that the average speed and maximum speed of the working day's cycle are significantly lower than the other cycles. However, speed values are notably higher on holidays. By analyzing the average positive acceleration and average negative acceleration, it is realized that the working days' cycle and the FTP-72 cycle share a strong resemblance. In summary, it is concluded that the driving cycle developed on working days is almost similar to the FTP-72 driving cycle, with a minor difference, particularly in speed values, which may be associated with the higher traffic observed in the working days' driving cycle.

After creating the driving cycles to evaluate the data, the error value is obtained for the parameters expressed on the main data and the final driving cycles. For this purpose, the RMSE and standard deviation are calculated through Equations (10), (11).

(10) $$RMSE=\sqrt{\frac{\sum_{i=1}^{n}{\left(\left(Main\_Data\_Parameter_{i}\right)-\left(DC\_Parameter_{i}\right)\right)}^{2}}{n}}$$

(11) $$Standard\_Deviation=\sqrt{\frac{\sum_{i=1}^{n}{\left(x_{i}-\mu \right)}^{2}}{n}},\mu =\frac{\sum_{i=1}^{n}x_{i}}{n}$$

$Main\_Data\_Parameter_{i}$ represent characteristic parameters of main data and $DC\_Parameter_{i}$ are characteristic parameters of driving cycles. In these equations, n is equal to the number of characteristic parameters and equal to 12. Table 6 demonstrates the RMSE and standard deviation of characteristic parameters. According to this table, the real-time collected data and the developed driving cycles in both holidays and working days modes exhibit similar variance and standard deviation values. To be more precise, the real-time and developed driving cycles display comparable behaviors, which supports the effectiveness of the proposed developed cycles. The standard RMSE values for driving cycles on holidays and working days are 1.321 and 12.029, respectively. Given that the range of parameters is between [0,100], this further validates the performance of the developed driving cycles [54].

**Table 6:** RMSE and standard deviation results of main and developed characteristic parameters.

Parameters	Main parameters _holidays	Developed parameters _Holidays	Main parameters _working days	Developed parameters _Working days
Percent of time driving (%)	98.56	97.34	86.60	91.68
Percent of time accelerating (%)	47.25	49.50	38.02	38.93
Percent of time cruising (%)	16.74	16.27	14.93	17.22
Percent of time decelerating (%)	34.57	31.56	33.65	35.52
Percent of time standing (%)	1.44	2.65	13.40	8.32
Average trip speed (km/h)	38.08	39.64	6.47	23.29
Average driving speed (km/h)	39.60	40.46	7.48	25.42
The standard deviation of speed (km/h)	42.48	43.16	30.56	28.30
Maximum speed(km/h)	80.89	80.89	21.36	54.00
Average positive acceleration (m/s^2^)	0.43	0.42	0.46	0.43
Average negative acceleration (m/s^2^)	−0.57	−0.62	−0.50	−0.46
The standard deviation of acceleration (m/s^2^)	0.65	0.86	0.60	0.59
Sum of Squares	11449.685	11333.002	6633.315	7747.415
Variance	954.140	944.417	552.776	645.618
Standard Deviation	30.889	30.731	23.511	25.409
RMSE	1.321		12.029	

### 3.3 Fuel consumption and emissions

Fuel consumption denotes the amount of fuel consumed by a vehicle to travel a certain distance at a particular speed [55]. Hydrocarbon, nitrogen oxides, and carbon monoxide are three exhaust emissions from vehicles with internal combustion engines, which are the primary causes of air pollution.

To determine fuel consumption and emissions, Advanced Vehicle Simulator (ADVISOR) software is employed. ADVISOR is a widely used simulation model for designing and analyzing advanced vehicle powertrains, such as conventional, hybrid, and electric vehicles [56]. ADVISOR utilizes drivetrain component performance to estimate fuel consumption and emissions on specific cycles, as well as maximum-effort acceleration capability [57]. This involves defining vehicle characteristics such as make, model, engine type, and transmission, as well as inputting specific parameters like vehicle weight, length, etc., as shown in Table 3. The developed driving cycles for specific dates are utilized to assess vehicle performance and emissions. To calculate fuel consumption, information about the fuel type is provided. Following the setup of all necessary parameters and running the simulation, fuel consumption and emissions data are obtained.

**Fig. 9:**
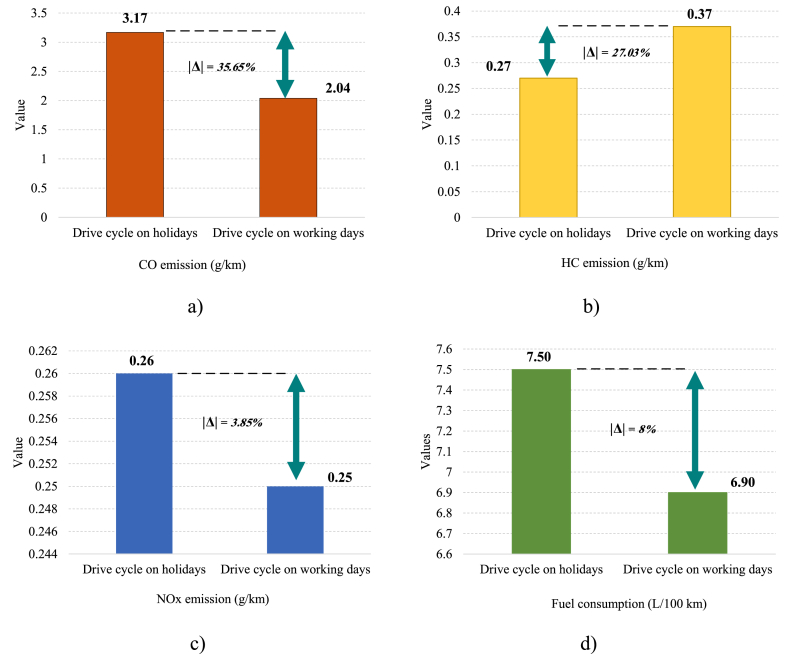
(a) CO emission, (b) HC emission, (c) NOx emission and (d) Fuel consumption values on both holidays and working days.

A comparison of real driving emissions performance, and fuel consumption between working days and holidays is shown in Fig. 9. Fig. 9a, 9.b, and 9.c demonstrate emissions of CO, HC, and NOx, respectively. Also, Fig. 9d represents fuel consumption. As it is apparent, the results show that the HC, CO, NOx, and fuel consumption values for both driving cycles on holidays and working days are different. On holidays, HC emission is about 0.27 g/km, which is 27.03 % lower than HC emission on working days. Nevertheless, CO emission, NOx emission, and fuel consumption on holidays are 35.65 %, 3.85 %, and 8 % higher compared to working days, respectively.

It is commonly believed that emissions and fuel consumption on holidays and weekends are lower than on weekdays [58]. However, the characteristics of holidays and hours of the day significantly impact the intensity of emissions and fuel consumption. For example, a study [28] showed that although NOx emissions around Beijing are higher on working days than on holidays and weekends, the highest amount of NOx is found in the weekend afternoons. Fuel consumption and emissions of HC, CO, and NOx are directly influenced by various driving parameters. Here is a general overview of how each emission is affected by different driving conditions.

1 Standing time: Higher standing time leads to increased fuel consumption and higher emissions of HC, NOx, and CO. However, compared to driving time, the amount of fuel consumption and emissions is considerably lower.
2 Speed: Higher speeds generally result in increased fuel consumption due to the engine working harder to maintain the speed. This leads also to higher emissions.
3 Acceleration: Rapid acceleration requires more fuel to be burned quickly, leading to higher fuel consumption and emissions of HC, CO, and NOx. Smooth and gradual acceleration can help reduce fuel consumption and emissions.
4 Deceleration: Deceleration, or the process of slowing down a vehicle, can impact fuel consumption and emissions. Sudden braking or hard deceleration can cause fuel wastage and higher emissions, as the engine has to work harder to maintain the desired speed. Smooth deceleration, such as using engine braking or coasting, can help reduce fuel consumption and emissions.

The impact of driving characteristic parameters on emissions and fuel consumption may vary in terms of their significance, potentially altering the overall outcome. For example, the emission rates of NOx and CO exhibit greater sensitivity to acceleration and speed. In slow zones and medium-speed areas, the values decrease with increasing absolute acceleration, whereas in high-speed zones, they rise with greater acceleration and speed [59]. The NOx and CO emissions are highest when both acceleration and speed reach their maximum levels, whereas the highest HC emission rate is typically observed in the zone with high acceleration and medium speed. During the tests conducted in a local driving cycle, it was observed that the acceleration mode exhibited the highest rates for CO and NOx, whereas the standing mode had the lowest values [60].

This is expected according to the characteristic parameters of working days and holidays shown in Table 4. According to the table, on holidays, speed and acceleration are generally higher compared to working days, leading to higher CO and NOx emissions. However, lower speed on working days result in higher HC emissions. Fuel consumption on weekdays influenced by lower driving time, lower speed, and lower accelerating time and was lower in comparison to holidays.

## 4 Conclusions

This research presents a novel methodological framework for designing driving cycles, which combines K-means clustering and PCA to identify sensitive parameters. Data was collected using a GPS logger in a private vehicle during a trip along a route selected using the AHP in Shiraz city, with recordings made on both holiday and working days. The GPS data was then analyzed to extract relevant features, which were computed separately for holiday and working days. The results of this analysis provide insights into the characteristics of driving cycles on both holiday and working days. According to these results.

• The Shiraz driving cycles' emission and fuel consumption values are found to be different on working days in comparison to holidays.
• All results are based on working day and holiday driving cycles and characteristics parameters such as speed, acceleration, driving time percentage, and etc.
• Fuel consumption on working days is about 8 % lower than on holidays due to the lower driving time (about 5.81 %) and lower average speed (almost 37.17 %).
• HC emissions are found to be about 27 % lower on holidays, due to the higher average speeds observed during this period, which consequently contribute to reduced HC emissions as compared to working days with relatively lower speeds.
• On the other hand, CO and NOx emissions are about 36 % and 4 % higher on holidays, respectively. This can be attributed to the higher average speeds and acceleration, experienced during holidays, which lead to increased fuel consumption and subsequently higher CO and NOx emissions.

Examining the relationship between date-specific factors, such as working days and holidays, can provide insights into their influence on fuel consumption and emissions in internal combustion engines. By analyzing the impact of these factors on driving patterns and characteristic parameters, researchers can better understand how they contribute to variations in HC, CO, and NOx emissions. These findings can help identify potential improvements in engine technologies, driving behavior, and transportation infrastructure, ultimately leading to enhanced efficiency and reduced environmental impacts for internal combustion engines. This study encourages the use of electric or hybrid cars in the city and suggests optimizing engines and catalysts to reduce fuel consumption and emissions. Additionally, it encourages further studies in other cities to gain a more comprehensive understanding of this issue.

## Funding

This research did not receive any specific funding.

## Ethical approval statement

The data collection of vehicles in this study was conducted by one author of this work, Elmira Bagheri, who has confirmed the use of the data for the publication, as also the first author of this manuscript. For the consent statement, first of all, the researchers explained the research objectives to the participants and the participation was voluntary in this survey. Moreover, all participants who fulfilled the questionnaire were informed that consent to participate in this study and publishing their data would be assumed.

## Data availability statement

The data that support the findings of this study will be available at the following GitHub link:

https://github.com/elmirabagheri/date_specific_analytics.

## CRediT authorship contribution statement

**Elmira Bagheri:** Writing – original draft, Validation, Software, Methodology, Investigation, Formal analysis, Conceptualization. **Masoud Masih Tehrani:** Writing – review & editing, Supervision, Formal analysis. **Mohammad Azadi:** Validation, Supervision, Formal analysis, Data curation. **Ashkan Moosavian:** Writing – original draft, Validation, Supervision, Methodology. **Sarah Sayegh:** Software, Data curation. **Mansour Hakimollahi:** Software, Data curation.

## Declaration of competing interest

The authors declare that they have no known competing financial interests or personal relationships that could have appeared to influence the work reported in this paper.

## References

[1] Wang L., Ma J., Zhao X., Li X. (2021). Development of a typical urban driving cycle for battery electric vehicles based on kernel principal component analysis and random forest. IEEE Access.

[2] Zhang Z., Liu H., Li Y., Ye Y., Tian J., Li J., Xu Y., Lv J. (2024). Research and optimization of hydrogen addition and EGR on the combustion, performance, and emission of the biodiesel-hydrogen dual-fuel engine with different loads based on the RSM. Heliyon.

[3] Ceper B.A., Yıldız M. (2017). Experimental investigation of performance and emissions of the SICAI-hybrid engine systems. Int. J. Hydrogen Energy.

[4] Lyu P., Wang P.S., Liu Y., Wang Y. (2021). Review of the studies on emission evaluation approaches for operating vehicles. J. Traffic Transp. Eng..

[5] Engelmann D., Zimmerli Y., Czerwinski J., Bonsack P. (2021). Real driving emissions in extended driving conditions. Energies.

[6] Kousoulidou M., Fontaras G., Ntziachristos L., Bonnel P., Samaras Z., Dilara P. (2013). Use of portable emissions measurement system (PEMS) for the development and validation of passenger car emission factors. Atmos. Environ..

[7] Tang T.-Q., Yi Z.-Y., Lin Q.-F. (2017). Effects of signal light on the fuel consumption and emissions under car-following model. Phys. A Stat. Mech. its Appl..

[8] Del Pero F., Delogu M., Pierini M. (2017). The effect of lightweighting in automotive LCA perspective: estimation of mass-induced fuel consumption reduction for gasoline turbocharged vehicles. J. Clean. Prod..

[9] Triantafyllopoulos G., Dimaratos A., Ntziachristos L., Bernard Y., Dornoff J., Samaras Z. (2019). A study on the CO2 and NOx emissions performance of Euro 6 diesel vehicles under various chassis dynamometer and on-road conditions including latest regulatory provisions. Sci. Total Environ..

[10] Pavlovic J., Fontaras G., Ktistakis M., Anagnostopoulos K., Komnos D., Ciuffo B., Clairotte M., Valverde V. (2020). Understanding the origins and variability of the fuel consumption gap: lessons learned from laboratory tests and a real-driving campaign. Environ. Sci. Eur..

[11] Teymoori M.M., Chitsaz I., Kashani N.A., Davazdah Emami M. (2023). Cold-start emission reduction of the gasoline-powered vehicle utilizing a novel method. Int. J. Engine Res..

[12] Weiss M., Paffumi E., Clairotte M., Drossinos Y., Vlachos T., Bonnel P., Giechaskiel B. (2017).

[13] Pielecha J., Skobiej K., Kurtyka K. (2021). Testing and evaluation of cold-start emissions from a gasoline engine in RDE test at two different ambient temperatures. Open Eng..

[14] Varella R.A., Duarte G., Baptista P., Villafuerte P.M., Sousa L. (2017). Analysis of the influence of outdoor temperature in vehicle cold-start operation following EU real driving emission test procedure. SAE Int. J. Commer. Veh..

[15] Gebisa A., Gebresenbet G., Gopal R., Nallamothu R.B. (2021). Driving cycles for estimating vehicle emission levels and energy consumption. Futur. Transp..

[16] Liu H., Zhao J., Qing T., Li X., Wang Z. (2021). Energy consumption analysis of a parallel PHEV with different configurations based on a typical driving cycle. Energy Rep..

[17] Shi Q., Liu B., Guan Q., He L., Qiu D. (2020). A genetic ant colony algorithm-based driving cycle generation approach for testing driving range of battery electric vehicle. Adv. Mech. Eng..

[18] Zhao X., Zhao X., Yu Q., Ye Y., Yu M. (2020). Development of a representative urban driving cycle construction methodology for electric vehicles: a case study in Xi’an. Transp. Res. Part D Transp. Environ..

[19] Williams R., Hamje H., Andersson J., Ziman P. (2018).

[20] Mafi S., Kakaee A., Mashadi B., Moosavian A., Abdolmaleki S., Rezaei M. (2022). Developing local driving cycle for accurate vehicular CO2 monitoring: a case study of Tehran. J. Clean. Prod..

[21] Bakibillah A.S.M., Kamal M.A.S., Tan C.P., Hayakawa T., Imura J.-I. (2019). Event-driven stochastic eco-driving strategy at signalized intersections from self-driving data. IEEE Trans. Veh. Technol..

[22] Bakibillah A.S.M., Kamal M.A.S., Tan C.P. (2020). 2020 59th Annual Conference of the Society of Instrument and Control Engineers of Japan (SICE).

[23] Bakibillah A.S.M., Kamal M.A.S., Tan C.P., Hayakawa T., Imura J. (2021). Fuzzy-tuned model predictive control for dynamic eco-driving on hilly roads. Appl. Soft Comput..

[24] Bakibillah A.S.M., Kamal M.A.S., Imura J., Mukai M., Yamada K. (2024). “Eco-Driving on Hilly Roads in a Mixed Traffic Environment: A Model Predictive Control Approach,”.

[25] Shahariar G.M.H. (2022). Impact of driving style and traffic condition on emissions and fuel consumption during real-world transient operation. Fuel.

[26] Du Y., Wu J., Yang S., Zhou L. (2017). Predicting vehicle fuel consumption patterns using floating vehicle data. J. Environ. Sci..

[27] Wang B., Shao C., Ji X. (2017). Influencing mechanism analysis of holiday activity–travel patterns on transportation energy consumption and emissions in China. Energies.

[28] Shang J., Zheng Y., Tong W., Chang E., Yu Y. (2014). Proceedings of the 20th ACM SIGKDD International Conference on Knowledge Discovery and Data Mining.

[29] Sriniwas A., Amal J., Nandagopalan C., Simhachalan A., Pandey N. (2011). A real world drive cycle for India. SAE Technical Paper.

[30] Abas M.A., Rajoo S., Abidin S.F.Z. (2018). Development of Malaysian urban drive cycle using vehicle and engine parameters. Transp. Res. Part D Transp. Environ..

[31] MacCarthy B.L., Wasusri T. (2002). A review of non‐standard applications of statistical process control (SPC) charts. Int. J. Qual. Reliab. Manag..

[32] Saaty T.L. (2014).

[33] Buchanan J., Phillip S., Daniel V. (1999). Project Ranking Using ELECTRE III, Department of Management Systems University of Waikato, Hamilton, New Zealand, Research Report Series.

[34] Ghanbari Fard R., Safavi A.A., Setoodeh P. (2017). The traffic flow effect modeling on the air pollution of Shiraz city. Environ. Sci..

[35] Yıldız M., Arslannur B., Aktürk A. (2022). SIMULATED EMISSIONS, AND FUEL CONSUMPTION OF INTERNAL COMBUSTION AND HYBRID ENGINE VEHICLES, IN ACTUAL DRIVING CYCLES OF IĞDIR PROVINCE: A CASE STUDY.

[36] Kazemi F.M., Samadi S., Poorreza H.R., Akbarzadeh-T M.-R. (2007). Fourth International Conference on Information Technology (ITNG'07), Las Vegas, NV, USA.

[37] Nayeb Yazdi M., Arhami M., Delavarrafiee M., Ketabchy M. (2019). Developing air exchange rate models by evaluating vehicle in-cabin air pollutant exposures in a highway and tunnel setting: case study of Tehran, Iran. Environ. Sci. Pollut. Res..

[38] Sahebi S., Nassiri H., de Winter J.C.F. (2019). Correlates of self-reported driving aberrations in Tehran: a study at the level of drivers and districts. Transp. Res. part F traffic Psychol. Behav..

[39] Rahmati M.H., Yousefi S.R. (2013). Demand estimation for the Iranian automobile industry. Q. Rev. Econ. Financ..

[40] Saleki A., Rezazade S., Changizian M. (2017). 2017 Iranian Conference on Electrical Engineering.

[41] Qaraati T., Movahed A.M., Azadi M., Moosavian A., Nikkhah M. (2020). 11th International Conference on Internal Combustion Engines and Oil, Tehran.

[42] Nyberg P., Frisk E., Nielsen L. (2016). Driving cycle equivalence and transformation. IEEE Trans. Veh. Technol..

[43] Jameel A.M., Al-Salami Q.H. (2023). Principal component analysis technique for finding the best applicant for a job. Cihan Univ. J. Humanit. Soc. Sci..

[44] Miri S.E., Azadi M., Pakdel S. (2022). Development of a duty cycle with K-means clustering technique for hydraulic steering in an instrumented TIBA vehicle. Transp. Eng..

[45] Zhao X., Yu Q., Ma J., Wu Y., Yu M., Ye Y. (2018). Development of a representative EV urban driving cycle based on a k-means and SVM hybrid clustering algorithm. J. Adv. Transp..

[46] Azadi M., Malekan A., Shahsavand A., Micallef D. (2023). Raw driving data of passenger cars considering traffic conditions in Semnan city. Exp. Results.

[47] Norbakyah J.S., Nordiyana M.I., Anida I.N., Ayob A.F., Salisa A.R. (2021). myBas driving cycle for Kuala Terengganu city. Int. J. Electr. Comput. Eng..

[48] Karimi G., Masih-Tehrani M., Pourbafarani Z. (2021). Development of wheel loader duty cycle using hybrid Markov chain and genetic algorithm. SAE Int. J. Commercial Vehicles.

[49] Fotouhi A., Montazeri-Gh M. (2013). Tehran driving cycle development using the k-means clustering method. Sci. Iran..

[50] Chugh S., Kumar P., Muralidharan M., Kumar M., Sithananthan M., Gupta A., Basu B., Malhotra R.K. (2012). Development of Delhi driving cycle: a tool for realistic assessment of exhaust emissions from passenger cars in Delhi. SAE Technical Paper.

[51] Qaraati T., Momeni Movahed A., Azadi M., Moosavian S.A. (2021). Comparison of support vector machine and K-means algorithms performance in extracting the real driving cycle of combined tehran-amol. Amirkabir J. Mech. Eng..

[52] Karunasingha D.S.K. (2022). Root mean square error or mean absolute error? Use their ratio as well. Inf. Sci..

[53] Barlow T.J., Latham S., McCrae I.S., Boulter P.G. (2009).

[54] Draper C., Reichle R., de Jeu R., Naeimi V., Parinussa R., Wagner W. (2013). Estimating root mean square errors in remotely sensed soil moisture over continental scale domains. Remote Sens. Environ..

[55] Champeecharoensuk A., Dhakal S., Chollacoop N., Phdungsilp A. (2024). Greenhouse gas emissions trends and drivers insights from the domestic aviation in Thailand. Heliyon.

[56] Masih-Tehrani M., Ebrahimi-Nejad S., Dahmardeh M. (2020). Combined fuel consumption and emission optimization model for heavy construction equipment. Autom. Constr..

[57] Wipke K.B., Cuddy M.R., Burch S.D. (Nov. 1999). Advisor 2.1: a user-friendly advanced powertrain simulation using a combined backward/forward approach. IEEE Trans. Veh. Technol..

[58] Long Y., Yoshida Y., Li Y., Gasparatos A. (2022). Spatial-temporal variation of CO2 emissions from private vehicle use in Japan. Environ. Res. Lett..

[59] Pignatta G., Balazadeh N. (2022). Hybrid vehicles as a transition for full e-mobility achievement in positive energy districts: a comparative assessment of real-driving emissions. Energies.

[60] Yang Y., Li T., Hu H., Zhang T., Cai X., Chen S., Qiao F. (2019). Development and emissions performance analysis of local driving cycle for small-sized passenger cars in Nanjing, China. Atmos. Pollut. Res..

